# Synchronous Succinate Dehydrogenase Subunit B (SDHB)-Deficient Gallbladder Paraganglioma and Periduodenal Well-Differentiated Neuroendocrine Tumor: A Case Report

**DOI:** 10.7759/cureus.97123

**Published:** 2025-11-17

**Authors:** Saejin Lee, Matt Paz, Arjun Gupta, Robben Schat, Samantha Woehrle, Byoung Uk Park

**Affiliations:** 1 Department of Laboratory Medicine, BML Laboratory, Daejeon, KOR; 2 Department of Laboratory Medicine and Pathology, University of Minnesota, Minneapolis, USA; 3 Division of Hematology, Oncology and Transplantation, University of Minnesota, Minneapolis, USA; 4 Department of Radiology, University of Minnesota, Minneapolis, USA; 5 Cancer Risk Management Program, MHealth Fairview, Minneapolis, USA

**Keywords:** gallbladder, germline variant, neuroendocrine tumor, paraganglioma, sdhb mutation, succinate dehydrogenase deficiency, synchronous tumors

## Abstract

Paragangliomas are extra-adrenal neuroendocrine neoplasms (NENs), and those arising in the gallbladder are exceedingly rare. Pathogenic variants in the succinate dehydrogenase (SDH) complex are well-established drivers of paragangliomas and pheochromocytomas, and have also been implicated in epithelial neuroendocrine tumors (NETs).

We report a 50-year-old woman with a likely pathogenic succinate dehydrogenase subunit B (SDHB*)* germline variant (p.D118Y, c.352G>T), who underwent surveillance imaging due to a family history of malignant carotid paraganglioma. Magnetic resonance imaging revealed a 2.5-cm retroperitoneal mass adjacent to the duodenum and pancreatic head, consistent on biopsy with a well-differentiated, low-grade NET showing loss of SDHB expression. Endoscopic evaluation incidentally identified a 1.2-cm enhancing nodule on the hepatic surface of the gallbladder wall. ^64^Cu-DOTATATE positron emission tomography/computed tomography (PET/CT) showed somatostatin receptor-positive uptake in both lesions. The patient underwent cholecystectomy with liver wedge resection and excision of the periduodenal mass. Histologic examination revealed two distinct tumors: a gallbladder paraganglioma showing a Zellballen pattern, positive for synaptophysin and chromogranin but negative for cytokeratin, and a periduodenal well-differentiated NET positive for cytokeratin AE1/AE3, synaptophysin, and chromogranin. Both lesions demonstrated complete loss of SDHB immunoreactivity, confirming cross-lineage SDH deficiency. The concurrent absence of SDHB expression in both tumors provides compelling phenotypic evidence supporting the pathogenicity of the germline SDHB variant, and reinforces the biologic coherence of the genotype-phenotype correlation. The postoperative course was uneventful, and follow-up imaging at eight months showed no recurrence. This unique case of synchronous SDHB-deficient gallbladder paraganglioma and periduodenal NET illustrates cross-lineage tumorigenesis within a single germline background and emphasizes the value of SDH immunohistochemistry and genotype-guided surveillance.

## Introduction

Paragangliomas are extra-adrenal neuroendocrine neoplasms (NENs) arising from autonomic paraganglia along parasympathetic and sympathetic chains [1]. In the gallbladder, paraganglioma is exceptionally rare, with only 24 cases reported to date [2,3]. Most cases were incidentally found after cholecystectomy, and no further treatment was needed when localized [2,3].

Pathogenic variants in the succinate dehydrogenase (SDH) complex are a well-established molecular driver of pheochromocytomas and paragangliomas [4]. In addition, SDH-deficient pancreatic neuroendocrine tumors (NETs) have been reported, extending the SDH-related tumor spectrum to epithelial NETs [5].

In this report, we present a gallbladder paraganglioma occurring synchronously with a periduodenal well-differentiated neuroendocrine tumor (WD-NET) in a patient with a likely pathogenic succinate dehydrogenase subunit B (SDHB) germline variant. To our knowledge, this case represents the first SDHB-associated gallbladder paraganglioma with a synchronous epithelial SDH-deficient NET, demonstrating cross-lineage SDH inactivation within a single germline background.

## Case presentation

A 50-year-old woman with a history of hypertension and a known likely pathogenic SDHB germline variant (p.D118Y, c.352G>T) was referred for routine surveillance whole-body, non-contrast magnetic resonance imaging (MRI). She had no prior cancer history, aside from fibrous dysplasia of the left femoral neck, resected 15 years earlier. Her family history was notable for malignant carotid paraganglioma in her mother, who also carries the familial SDHB variant.

Screening whole-body MRI identified a 2.5-cm, well-circumscribed retroperitoneal mass adjacent to the duodenum and pancreatic head. Endoscopic ultrasound-guided fine-needle aspiration of this lesion revealed tumor cells morphologically and immunohistochemically consistent with a well-differentiated, low-grade NET, positive for synaptophysin, pancytokeratin, and insulinoma-associated protein 1 (INSM1), and with a Ki-67 index of <2%. Loss of SDHB expression raised the possibility of an SDHB-deficient NET.

**Figure 1:**
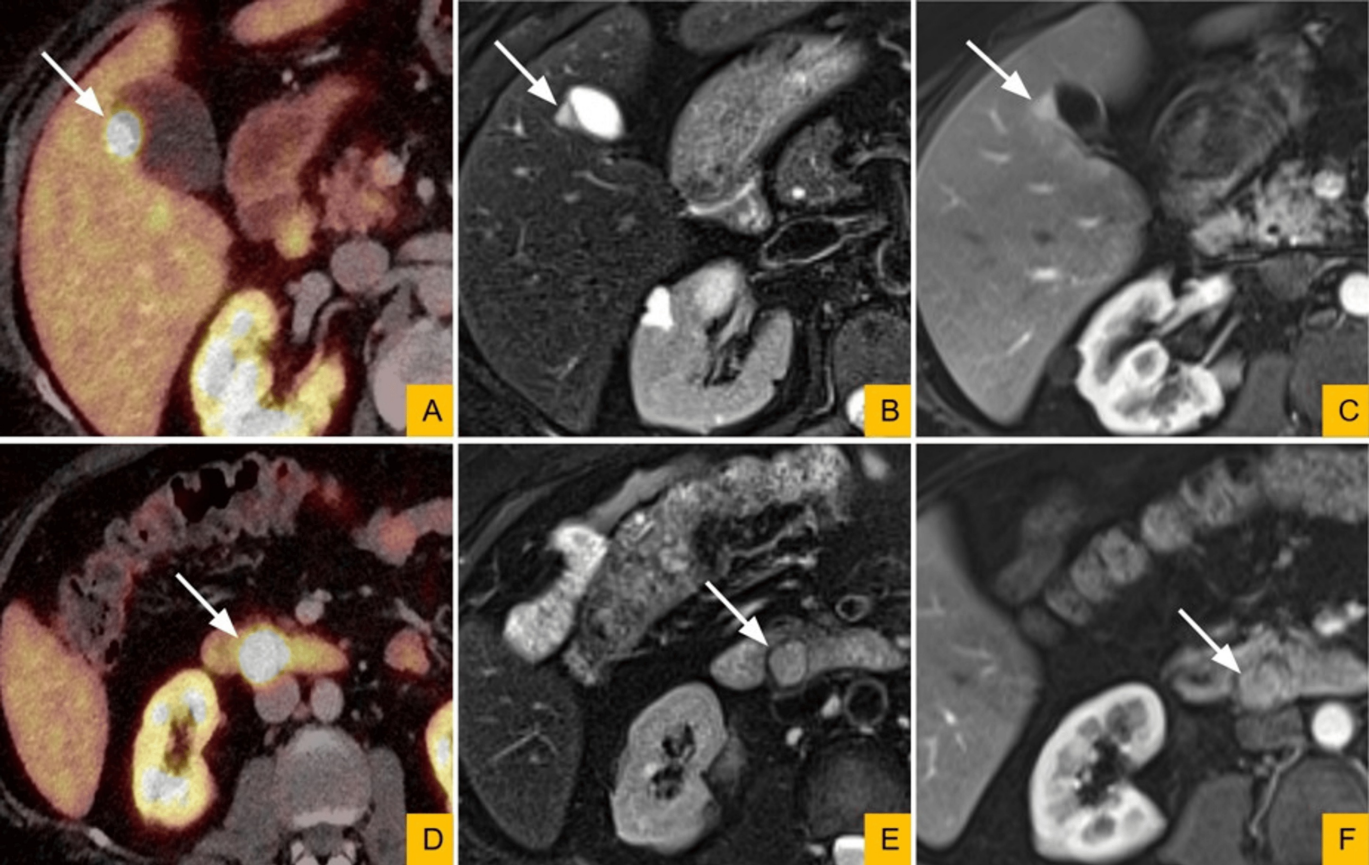
Synchronous Gallbladder and Periduodenal Lesions Demonstrated by DOTATATE PET/CT and MRI. (A, D) Axial DOTATATE PET/CT images demonstrate a 1.1-cm gallbladder wall lesion and a 2.5-cm periduodenal lesion, both with DOTATATE avidity, indicating somatostatin receptor positivity (white arrows). (B, E) Dedicated abdominal MRI demonstrates corresponding, well-circumscribed lesions that are hyperintense on axial T2-weighted images (white arrows). (C, F) Lesions are arterially enhancing on T1-weighted post-contrast images (white arrows). PET/CT, positron emission tomography/computed tomography; MRI, magnetic resonance imaging

During endoscopic ultrasound evaluation of the periduodenal lesion, an incidental 1.2-cm nodule was noted on the hepatic surface of the gallbladder wall. Subsequent whole-body ^64^Cu-DOTATATE positron emission tomography-computed tomography (PET/CT) revealed somatostatin receptor-positive lesions along the lateral gallbladder wall and posterior to the second portion of the duodenum. A dedicated contrast-enhanced abdominal MRI demonstrated both tumors to be well-circumscribed, T2-hyperintense, and avidly arterially enhancing, with the gallbladder lesion measuring 1.1 cm and the periduodenal lesion measuring 2.5 cm (Figure 1). The distinction between synchronous primary tumors and a single primary tumor with metastasis could not be established. Given these findings, cholecystectomy with wedge resection of segment 5 of the liver, and excision of the periduodenal mass were performed.

Intraoperatively, the gallbladder appeared grossly normal, but palpation revealed a 1-cm, firm nodule along the lateral superior hepatic surface. Additionally, a firm, white, exophytic lesion was identified in the pancreatic-duodenal groove at the junction of the second and third portions of the duodenum. Intraoperative ultrasound showed a homogeneous, well-encapsulated mass without invasion and without a clear origin from either the duodenum or pancreas. The lesion was circumferentially dissected and enucleated from the peripancreatic fat. Gross examination showed a 1.4-cm, tan-white, encapsulated mass within the gallbladder adventitia, contiguous with the resected liver wedge. The pancreatic groove mass measured 2.6 cm, tan-brown, and ovoid, with a friable, white-yellow cut surface.

**Figure 2:**
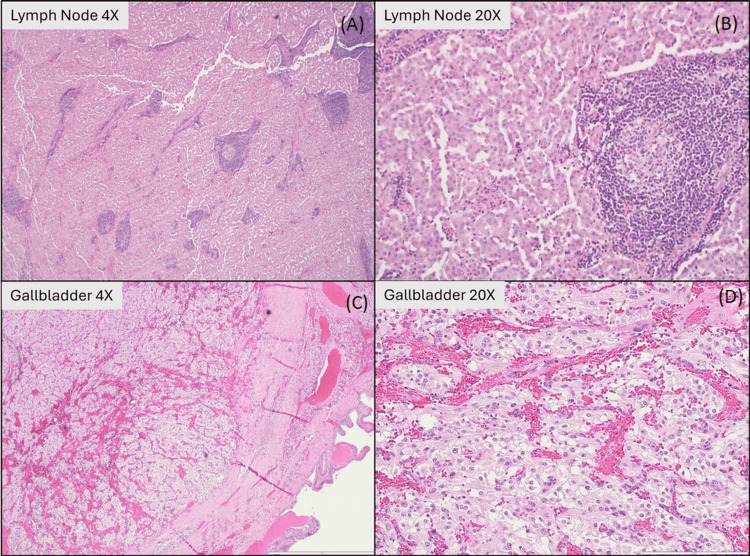
Histologic features of the periduodenal well-differentiated neuroendocrine tumor (WD-NET) replacing lymph nodes, and of the gallbladder paraganglioma. (A) Periduodenal mass, low-power view showing a well-circumscribed neuroendocrine neoplasm composed of nests and trabeculae within a delicate fibrovascular stroma (hematoxylin and eosin (H&E), original magnification ×4). (B) Higher-power view demonstrating uniform neuroendocrine cells arranged in an organoid pattern, consistent with a well-differentiated neuroendocrine tumor (H&E, ×20). (C) Gallbladder specimen, low-power view showing a non-epithelial neuroendocrine neoplasm centered in the wall with a nested (Zellballen) architecture and intact overlying mucosa (H&E, ×4). (D) Higher-power view demonstrating classic nested architecture with a delicate capillary network, compatible with paraganglioma (H&E, ×20).

Histological evaluation of the gallbladder lesion demonstrated epithelioid cells arranged in a classic Zellballen pattern, separated by vascular stroma (Figure 2). The lesion was located within the perimuscular tissue, 0.1 cm from the liver parenchymal margin. The periduodenal mass showed replacement of two lymph nodes by monotonous, epithelioid cells arranged in an organoid pattern (Figure 2).

**Figure 3:**
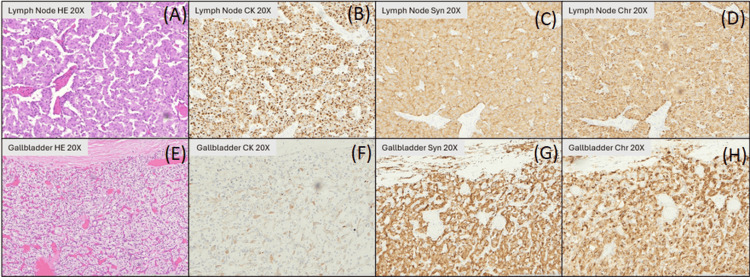
Immunohistochemical stains of the periduodenal well-differentiated neuroendocrine tumor (WD-NET) and the gallbladder paraganglioma (all panels, original magnification ×20). (A) Periduodenal tumor on hematoxylin and eosin (H&E) showing an organoid pattern with trabecular growth. (B) Pancytokeratin (AE1/AE3) highlighting diffuse cytoplasmic positivity in tumor nests, supporting epithelial differentiation. (C) Synaptophysin with diffuse cytoplasmic staining. (D) Chromogranin A demonstrating granular cytoplasmic positivity, consistent with a WD-NET. (E) Gallbladder lesion on H&E showing classic nested (Zellballen) architecture. (F) Pancytokeratin (AE1/AE3) negative in tumor cells, supporting a non-epithelial neuroendocrine neoplasm. (G) Synaptophysin with diffuse strong staining in chief cells. (H) Chromogranin A with diffuse granular cytoplasmic positivity, compatible with paraganglioma.

**Figure 4:**
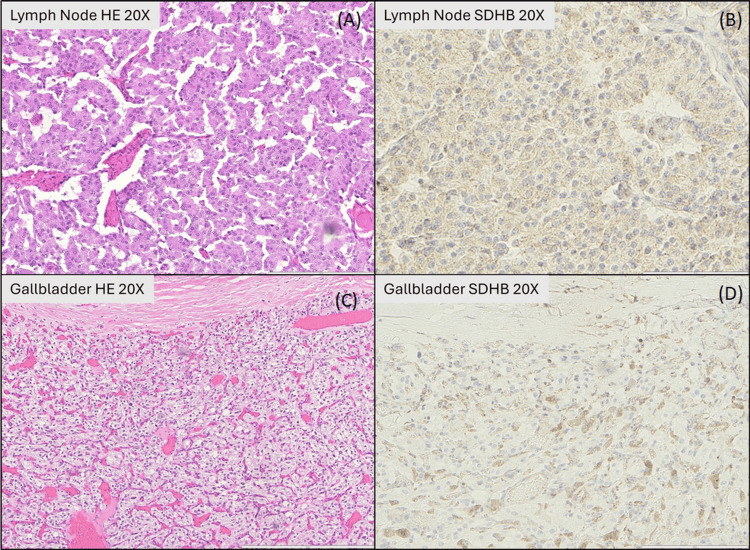
Histologic and succinate dehydrogenase subunit B (SDHB) immunohistochemical features of the periduodenal well-differentiated neuroendocrine tumor (WD-NET) and the gallbladder paraganglioma. (A) Periduodenal WD-NET in a regional lymph node, showing organoid nests and trabeculae of monotonous epithelioid cells with moderate eosinophilic cytoplasm (hematoxylin and eosin (H&E), ×20). (B) SDHB immunostain on the same lymph node demonstrates complete absence of granular cytoplasmic staining in tumor cells, consistent with SDHB loss (SDHB, ×20). Stain performance verified on an external positive control slide (not shown). (C) Gallbladder lesion, composed of a non-epithelial neuroendocrine neoplasm with classic paraganglioma morphology, including Zellballen nests of epithelioid cells separated by delicate fibrovascular stroma, centered in the gallbladder wall (H&E, ×20). (D) SDHB immunostain on the gallbladder lesion also shows complete loss of cytoplasmic staining in neoplastic cells, confirming SDHB-deficient paraganglioma (SDHB, ×20). Stain performance verified on an external positive control slide (not shown).

Immunohistochemistry of the gallbladder lesion revealed positivity for synaptophysin and chromogranin, with negativity for cytokeratin AE1/AE3, confirming the diagnosis of paraganglioma (Figure 3). In contrast, the periduodenal mass showed positivity for cytokeratin AE1/AE3, synaptophysin, and chromogranin, confirming WD-NET (Figure 3). Notably, SDHB expression was lost in both tumors, supporting cross-lineage SDH deficiency (Figure 4). This uniform loss of SDHB expression across two histologically distinct neoplasms strengthens the interpretation that the germline SDHB variant is pathogenic, linking the molecular alteration to its phenotypic manifestation.

Postoperative recovery was uneventful. The patient had no clinical evidence of hormone overproduction before, during, or after surgery. Plasma metanephrine levels were within normal limits pre- and postoperatively. Surveillance cross-sectional imaging at eight months showed no evidence of disease. She will continue annual whole-body imaging, alternating MRI and DOTATATE PET, to surveil for recurrence and new primaries.

## Discussion

Paragangliomas are NENs that arise from paraganglia derived from the neural crest and show a high rate of hereditary predisposition in contemporary series [1,4]. These NENs that arise in the adrenal gland are referred to as pheochromocytomas, whereas extra-adrenal paragangliomas occur along the sympathetic and parasympathetic chains; both are classified as non-epithelial NENs [1,6]. In parallel, epithelial NENs are organized as WD-NETs and poorly differentiated neuroendocrine carcinomas, with grading based on mitotic count and Ki-67 index, underscoring that paragangliomas and epithelial NETs are biologically distinct entities [6].

Paragangliomas can be categorized by their autonomic association. Parasympathetic tumors, typically centered in the head and neck around the carotid body, jugular foramen, and other skull-base sites, are usually non-secretory and often asymptomatic [1,4]. Sympathetic tumors predominate along the para-aortic and paravertebral chains of the thoracoabdominal region, frequently secrete catecholamines, and manifest with adrenergic symptoms such as palpitations, diaphoresis, and paroxysmal hypertension [1,4]. Familial disease accounts for a substantial minority of cases, most commonly due to germline variants in the SDH complex and other susceptibility genes, such as Von Hippel-Lindau (VHL), rearranged during transfection (RET), fumarate hydratase (FH), and neurofibromin 1 (NF1) [1,4].

Gallbladder paraganglioma is exceptionally rare. There are 24 cases described worldwide since the 1970s through reviews and case series. Most were tumors discovered incidentally on microscopic examination after cholecystectomy performed for biliary colic, cholelithiasis, or nonspecific right upper quadrant pain [2,3,7-12]. Patients are commonly middle-aged, often female, and the lesions are typically minute nodules in the adventitia or gallbladder wall, frequently accompanied by chronic cholecystitis or gallstones [2,3,8,9,11,12]. Functional catecholamine secretion is uncommon in this location, and perioperative hemodynamic lability has seldom been documented [2,3].

Histologically, gallbladder paraganglioma reproduces the classic Zellballen pattern in a richly vascular stroma with S100-positive sustentacular cells, diffuse chromogranin A and synaptophysin expression, and lack of keratin reactivity, helping to distinguish it from epithelial NETs of the gallbladder and adjacent biliary tract [2,3]. Complete excision by cholecystectomy has been the dominant treatment in the literature, and short- to intermediate-term outcomes have been favorable without local recurrence or metastasis; nevertheless, individualized follow-up is reasonable given the behavior of extra-adrenal paragangliomas at other intra-abdominal sites and the not-infrequent hereditary background [2-4].

The periduodenal lesion in our case demonstrated replacement of two lymph nodes, a finding that merits careful diagnostic consideration. Differential possibilities include a rare primary lymph-node NET, metastasis from an occult primary, or a primary soft-tissue NET with secondary nodal involvement. In this patient, the absence of an identifiable primary source, the well-circumscribed architecture, and the very low proliferative index (Ki-67 <2%) collectively favor a primary soft-tissue origin with nodal replacement rather than metastatic disease. This distinction carries clinical relevance, as even low-grade SDH-deficient NETs may display unpredictable biologic behavior, underscoring the need for long-term imaging surveillance [5,13]. The periduodenal tumor was regarded as a primary soft-tissue NET rather than a metastasis, given its well-circumscribed nature, absence of continuity with the duodenal or pancreatic parenchyma, absence of a primary gastrointestinal source, and lack of other primary sites on imaging.

Hereditary susceptibility and synchronous NENs have been described. A gallbladder paraganglioma associated with a pathogenic variant in the succinate dehydrogenase subunit D (SDHD) gene has been reported, identified by a fluorodopa PET, which demonstrated unexpected tracer avidity in the gallbladder region during surveillance of hereditary disease [13]. Another report documented synchronous detection of gallbladder paraganglioma and a pancreatic WD-NET in a carrier of a pathogenic succinate dehydrogenase subunit A (SDHA) variant identified through somatostatin receptor imaging and genetic testing [14]. Importantly, the spectrum of SDH-deficient neoplasia extends beyond paraganglia: pancreatic WD-NETs lacking SDHB expression have been demonstrated and are now recognized within the SDH-related tumor spectrum [5]. These observations support a genotype-aware approach to imaging, pathologic work-up, and surveillance in patients and families carrying variants in the SDH complex [1,15].

The patient is heterozygous for the SDHB missense variant c.352G>T (p.Asp118Tyr), which is classified as likely pathogenic in the ClinVar database. Tumor tissue was not submitted for sequencing; therefore, loss of heterozygosity (LOH) could not be directly confirmed. Nonetheless, the complete loss of SDHB immunoreactivity in both the gallbladder paraganglioma and the periduodenal WD-NET provides strong functional evidence of biallelic inactivation of SDHB, consistent with the expected two-hit mechanism in SDH-deficient neoplasia.

Compared with the previously reported SDHA-related case [14], SDHB variants are generally associated with higher malignant potential and a greater likelihood of metastasis. Recognizing this distinction underscores the need for heightened clinical vigilance and genotype-tailored surveillance strategies in SDHB carriers.

To our knowledge, this case represents the first SDHB-associated gallbladder paraganglioma with synchronous epithelial SDH-deficient NET, demonstrating cross-lineage SDH inactivation within a single germline background. In summary, the present case of synchronous gallbladder paraganglioma and a periduodenal WD-NET in a confirmed SDHB germline carrier adds two points. First, it complements the prior SDHA-related report by documenting a distinct genotype with the same cross-lineage pattern, namely, non-epithelial paraganglioma and epithelial NET arising in the same individual [5,14]. Second, it operationalizes the biologic link by showing loss of SDHB expression in the epithelial tumor on immunohistochemistry, which aligns with the World Health Organization concept of SDH-deficient NENs, and supports surveillance strategies that consider both paragangliomas and epithelial NETs in carriers of SDH variants [1,6,15].

## Conclusions

Gallbladder paraganglioma is exceedingly rare and usually non-secretory, most often an incidental finding at cholecystectomy. We report synchronous gallbladder paraganglioma and a periduodenal WD-NET in an SDHB germline carrier, with loss of SDHB in the epithelial tumor, confirming cross-lineage SDH deficiency. This observation supports a genotype-aware approach that emphasizes the role of SDH immunohistochemistry, and individualized surveillance encompassing both paragangliomas and epithelial NETs. This report is limited by the absence of functional SDH enzymatic testing and the relatively short duration of follow-up. Continued longitudinal surveillance is ongoing.
